# Atorvastatin-Associated Eosinophilic Spongiosis: A Case Report and a Relevant Literature Review of Dermatological Manifestations

**DOI:** 10.7759/cureus.61071

**Published:** 2024-05-25

**Authors:** Feras Al-Moussally, Brittany M Thompson, Omar M Masarweh, Neel Shah, Sudeep Gaudi, Jorge Restrepo

**Affiliations:** 1 Internal Medicine, University of Central Florida College of Medicine, Orlando, USA; 2 College of Medicine, University of Central Florida, Orlando, USA; 3 Internal Medicine, University of Central Florida, Kissimmee, USA; 4 College of Medicine, University of Central Florida College of Medicine, Orlando, USA; 5 Dermatopathology, James A. Haley Veterans' Hospital, Tampa, USA; 6 Internal Medicine, Orlando Veterans Affairs Medical Center, University of Central Florida, Orlando, USA

**Keywords:** mellitus, eosinophilic, eosinophilic spongiosis, diabetes, atorvastatin

## Abstract

Atorvastatin, a widely prescribed 3-hydroxy-3-methyl-glutaryl-coenzyme A reductase inhibitor (HMG-CoA reductase inhibitor), is associated with various adverse effects, including many dermatologic manifestations. We present the case of a 73-year-old man who developed eosinophilic spongiosis shortly after initiating atorvastatin therapy, an adverse effect which to our knowledge has not yet been reported in association with atorvastatin. Our investigation explores the clinical and histopathologic characteristics of eosinophilic spongiosis induced by atorvastatin, delving into potential mechanisms behind statin-induced eosinophilia. A literature review, focusing on atorvastatin's dermatological side effects, revealed a limited number of relevant studies, emphasizing the scarcity of documented cases. Our aim is to raise awareness of eosinophilic spongiosis as a potential side effect of atorvastatin, emphasizing its impact on patients' quality of life. This case prompts further research into the mechanisms underlying such dermatologic reactions, contributing to a better understanding of atorvastatin's diverse adverse effects.

## Introduction

Atorvastatin, a powerful lipid-lowering agent, is one of the most commonly prescribed drugs in the United States. As such, it is important for physicians to be aware of its potential adverse effects. Some of the more common side effects include gastrointestinal upset, myalgia and arthralgia, and alterations in liver function tests as well as numerous cutaneous manifestations [1]. The most common dermatologic side effects are pruritus and an eczematous skin rash [2]. Eosinophilia has been linked to atorvastatin, though not in the form of eosinophilic spongiosis according to current literature [3]. According to the Food and Drug Administration Adverse Events Reporting System, there have been 3,993 cases of atorvastatin-induced dermatologic effects, the most common of which are pruritus and rash [4].

## Case presentation

Materials and methods

We performed an independent literature review of existing literature relating to atorvastatin’s dermatological side effects. PubMed was the main source in searching for relevant literature. Inclusion criteria for our search included the use of atorvastatin, inclusion of dermatological side effects, and proof in the form of histopathologic evidence of the reported reaction. Exclusion criteria included literature relating to other statins and non-dermatological adverse reactions to atorvastatin. A relevant literature review yielded 357 results. Of those, 20 results were relevant, full length, and in English and therefore met inclusion criteria. For our search, relevance was defined as meeting all inclusion criteria and no exclusion criteria.

A 73-year-old man with a history of tobacco use disorder, hyperlipidemia, obstructive sleep apnea, hypothyroidism, and prostate cancer status post prostatectomy presented to the hospital with worsening rash and swelling for four days.

**Figure 1:**
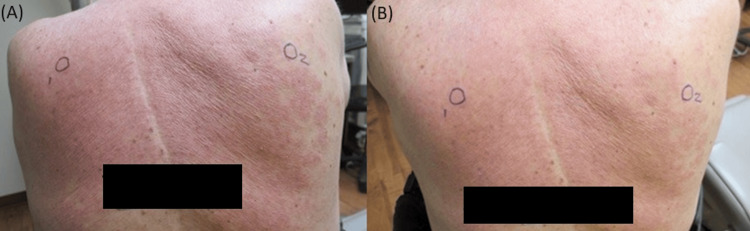
Erythematous scaling blanchable plaques on the back after re-exposure to atorvastatin Two pictures of the patient's back were taken on the same day from different angles.

**Figure 2:**
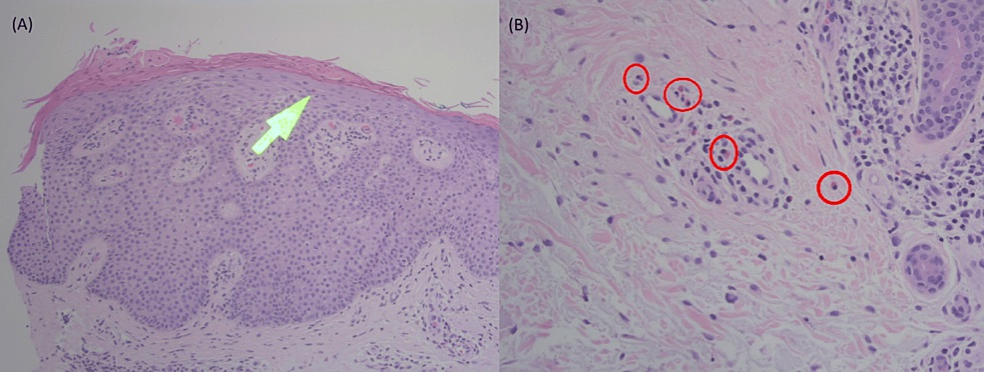
(A) Punch biopsy at 100x showing parakeratosis with intermixed serum and subjacent spongiosis and acanthosis. An eosinophil (arrow) in the dermis; (B) Punch biopsy at 200x showing multiple eosinophils in the dermal inflammatory infiltrate (circles).

The patient reported starting daily oral atorvastatin approximately one year ago. He reported the development of a severe diffuse rash one day after initiation. The rash immediately improved after discontinuation the next day, and he was never re-exposed to statins until the day of his admission. Since atorvastatin exposure, the patient has had recurrent spontaneous exacerbations of the same rash. Physical examination revealed erythematous scaling blanchable plaques on the back, shoulders, arms, and mid-chest (Figures 1A-1B). He was referred to a dermatologist where a punch biopsy showed parakeratosis, spongiosis, and acanthosis overlying a superficial and mid, perivascular, predominantly lymphohistiocytic infiltrate containing several eosinophils, with rare eosinophils involving the epidermis (Figures 2A-2B). Periodic Acid-Schiff (PAS) with diastase (PAS-D) stain did not show significant basement membrane changes or pathogenic fungal elements. Immunofluorescence was negative for IgG, IgM, IgA, and C3 deposits.

The patient was admitted to the hospital where he was started on intravenous diphenhydramine 25mg and methylprednisolone 125mg. His rash improved but did not completely resolve and he was discharged on oral diphenhydramine 25mg and topical fluocinonide with plans to follow up with his dermatologist.

## Discussion

Atorvastatin is commonly prescribed; therefore, it is important that prescribers be aware of potential adverse effects. The known dermatological adverse effects of atorvastatin are outlined in Table 1. Atorvastatin has been linked to numerous conditions that involve eosinophilia. These include eosinophilic fasciitis as well as Drug Reaction with Eosinophilia and Systemic Symptoms (DRESS syndrome) [4,5]. These syndromes can significantly impact the quality of life of a patient. The mechanism by which atorvastatin causes eosinophilia remains unknown [3].

**Table 1:** Known dermatological adverse effects of atorvastatin MCC: Merkel cell carcinoma

Adverse Effect	Presentation	Citation
Eosinophilic Fasciitis	Symmetrical painful swelling and induration of the extremities and disabling joint contractures	[5]
Drug Reaction with Eosinophilia and Systemic Symptoms	Febrile skin rash, facial edema, polymorphic lesions, oral mucosal involvement, fever, abdominal pain, diarrhea, polyarthralgia, and adenomegaly	[6]
Merkel Cell Carcinoma	The standardized incidence ratio for MCC was 3.16 in patients taking statins under the age of 60, typically presents as a single painless violet lump on sun-exposed skin, considered a neuroendocrine carcinoma of the skin	[7]
Phototoxic Effects	Edematous actinic erythema following sun exposure	[8]
Dermatomyositis/Polymyositis	Various presentations including endomysial and perimysial T-cell infiltration with muscular degeneration, myosis with atrophic myocytes, Reynaud’s, and heliotrope rash	[9,10]
Systemic Lupus Erythematosus/Subacute Cutaneous Lupus Erythematosus	Various presentations including interface dermatitis or scaly annular lesions along with fatigue, myalgia, polyarthralgia, leukopenia, focal segmental glomerulonephritis, and Raynaud's syndrome	[9,11]
Worsening of Psoriasis	Increase in erythematous scaling plaques with regular margins, increased itching, worsening of existing plaques	[12-14]
Lichenoid Drug Eruption	Purple scaling plaques with a dense band-like inflammatory infiltrate composed of lymphocytes in the upper dermis and along the dermoepidermal junction	[15]
Linear IgA Bullous Dermatosis	Autoimmune skin blistering with circulating IgA antibodies binding the basement membrane zone	[16]
Steven Johnson’s Syndrome/Toxic Epidermal Necrolysis	Large areas of erythema, blisters, epidermal exfoliation, and multi-site mucositis, often with systemic dysfunction	[17]

There is a proposed anti-inflammatory impact of statins in patients with asthma, which makes the eosinophilia with atorvastatin more surprising [18]. In these patients, statins are believed to decrease airway eosinophil recruitment for an anti-inflammatory effect. Fluvastatin and lovastatin have been seen to inhibit the adhesion of human eosinophils to recombinant human intracellular adhesion molecule-1. Fluvastatin and pravastatin have been observed to reduce the expression of intracellular adhesion molecule-1 itself [19]. NF-kB inhibition, which enhances eosinophil apoptosis, is also an effect of simvastatin [18].

However, in a mouse study where tracheal epithelial cells were treated with atorvastatin, there was induced expression of multiple chemokines such as eotaxin-1, which attracts eosinophils, as well as MCP-1, MCP-2, and MCP-3. These studies show that atorvastatin may behave differently than other common statins when it comes to inflammation and accumulation of eosinophils [20].

Eosinophilic spongiosis is a histopathological finding characterized by intraepidermal eosinophils and seen in numerous dermatological conditions. It often indicates the early stages of autoimmune bullous dermatosis. However, it can also be a finding in other inflammatory skin disorders such as spongiotic dermatitis and contact, atopic, or nummular dermatitis. Immunofluorescence studies can be used to confirm and distinguish between these disorders, which are often present without concomitant vesicles or blisters when eosinophilic spongiosis is present [21].

Atorvastatin-induced eosinophilic spongiosis could be a concern as it may be the initial finding for developing a more severe syndrome like those listed above. Treatment of atorvastatin-induced dermatological symptoms involves withdrawal of the offending agent as needed symptomatic treatment with other medications, such as topical or oral steroids.

It is unclear whether this effect is host-dependent and whether this represents a class effect to all statins or is specific to atorvastatin. This is important as the use of statins has strong evidence in primary and secondary prevention of atherosclerotic cardiovascular disease as well as first-line treatment for hyperlipidemia. Due to the debilitating nature of the reaction in our patient, he was not changed to a different statin and resorted to non-statin lipid-lowering agents. Fortunately, advances in other effective therapeutic options exist with great efficacy in lowering cholesterol and reducing cardiovascular risk.

Finally, this is simply a correlation effect noted between atorvastatin use and the development of eosinophilic spongiosis as there is no way to assure a causal relationship. However, the temporal association as well as the resolution of the rash temporally correlates.

## Conclusions

Statins have been associated with numerous dermatological side effects, ranging from mild to severe and possibly life-threatening. In this case, we present eosinophilic spongiosis as a side effect of atorvastatin use, which, from an expansive literature review, has not been previously reported. Due to their widespread use, prescribers must be aware of all potential side effects of statins in order to ensure the safety of their patients. The mechanism of eosinophilic spongiosis is unclear; therefore, further research is needed. Until then, withdrawal of the offending agent and the use of steroids seems to be a safe and effective treatment option.
